# The Acuity and Manipulability of the ANS Have Separable Influences on Preschoolers’ Symbolic Math Achievement

**DOI:** 10.3389/fpsyg.2018.02554

**Published:** 2018-12-11

**Authors:** Ariel Starr, Rachel C. Tomlinson, Elizabeth M. Brannon

**Affiliations:** ^1^Department of Psychology, University of California, Berkeley, Berkeley, CA, United States; ^2^Department of Psychology, University of Michigan, Ann Arbor, MI, United States; ^3^Department of Psychology, University of Pennsylvania, Philadelphia, PA, United States

**Keywords:** approximate number system, numerical cognition, math cognition, cognitive development, symbolic math

## Abstract

The approximate number system (ANS) is widely considered to be a foundation for the acquisition of uniquely human symbolic numerical capabilities. However, the mechanism by which the ANS may support symbolic number representations and mathematical thought remains poorly understood. In the present study, we investigated two pathways by which the ANS may influence early math abilities: variability in the acuity of the ANS representations, and children’s’ ability to manipulate ANS representations. We assessed the relation between 4-year-old children’s performance on a non-symbolic numerical comparison task, a non-symbolic approximate addition task, and a standardized symbolic math assessment. Our results indicate that ANS acuity and ANS manipulability each contribute unique variance to preschooler’s early math achievement, and this result holds after controlling for both IQ and executive functions. These findings suggest that there are multiple routes by which the ANS influences math achievement. Therefore, interventions that target both the precision and manipulability of the ANS may prove to be more beneficial for improving symbolic math skills compared to interventions that target only one of these factors.

## Introduction

Math ability when a child first enters schooling is the strongest predictor of later math and overall academic achievement (Duncan et al., 2007). However, there is variation in math ability across the population, and such variation is already present even before children first begin formal schooling (e.g., Libertus et al., 2011; Mazzocco et al., 2011; vanMarle et al., 2014). Many cognitive and socioeconomic factors are known to contribute to individual differences in math achievement. One of these factors is an evolutionarily ancient system for representing approximate quantities. Although humans use linguistic symbols to represent number, we also possess a system for representing number in an approximate, non-symbolic fashion. This system, termed the approximate number system (ANS), emerges independent of exposure to language or formal schooling and is present in a wide variety of non-human species, and emerges early in human development (e.g., Gallistel and Gelman, 1992; Dehaene, 1997; Hubbard et al., 2008).

The ANS is frequently hypothesized to be a cognitive foundation for symbolic math abilities. Lending support to this view is the finding that the acuity of the ANS, typically measured by an individual’s ability to compare two arrays of dots, correlates with symbolic math achievement throughout the lifespan (see Chen and Li, 2013 for review; Fazio et al., 2014; Schneider et al., 2017). Importantly, ANS acuity prior to the beginning of formal math instruction is predictive of later math achievement (Mazzocco et al., 2011; Libertus et al., 2013; Starr et al., 2013b; vanMarle et al., 2014). These studies suggest that the precision of approximate number representations may contribute to children’s acquisition of symbolic math principles and influence symbolic math performance throughout the lifespan.

Although many studies have focused on the link between ANS acuity and math achievement, relatively less attention has been paid to children’s ability to manipulate approximate numerical quantities. Beyond simply representing quantities, the ANS enables infants (McCrink and Wynn, 2004), preschoolers (Barth et al., 2005, 2006; Gilmore et al., 2010), and monkeys (Cantlon and Brannon, 2007) to perform approximate arithmetic operations without the use of symbols or formal training. The ANS has even been shown to contribute to algebraic problem solving in preschool-aged children (Kibbe and Feigenson, 2015). Therefore, the manipulability of the ANS may form a basis for the acquisition the basic arithmetic principles that underlie symbolic math. In support of this view, children’s approximate arithmetic performance at the beginning of kindergarten is predictive of their symbolic math achievement at the end of the academic year (Gilmore et al., 2010). Furthermore, practicing non-symbolic arithmetic in both preschool-aged children and adults leads to improvements in their symbolic arithmetic performance (Park and Brannon, 2013, 2014; Hyde et al., 2014; Park et al., 2016). Therefore, children who are more adept at manipulating approximate quantities in arithmetic operations may also be more adept at symbolic arithmetic because of the overlap in cognitive processes required by both forms of arithmetic. As a result of this overlap, it may be not only the *precision* of ANS representations that influences symbolic math achievement but also the *manipulability* of ANS representations.

However, though previous work suggests that the precision and manipulability of the ANS both contribute to symbolic math achievement, it is currently unknown whether these are separable factors. In other words, do children with more precise ANS representations necessarily also more adept at manipulating approximate quantities in arithmetic operations? If this is the case, then we would expect ANS manipulability to mediate the relation between ANS acuity and symbolic math achievement. Alternatively, if ANS acuity and manipulability are distinct, we would expect both factors to contribute unique variance to children’s early symbolic math performance.

In the present research, we explicitly tested how ANS acuity and manipulability each contribute to symbolic math achievement in preschool-aged children. We focused on preschool-aged children because they have not yet started formal schooling, so they have not yet been exposed to formal symbolic math education. Thus, we could assess how different aspects of children’s intuitive sense of number relate to their symbolic math proficiency. Children were tested with a non-symbolic numerical comparison task to assess ANS acuity, a non-symbolic approximate addition task to assess ANS manipulability, and a standardized symbolic math test. In addition, children performed a general IQ test and a subset of children performed an executive functions task^1^ in order to control for domain-general factors that also contribute to math achievement.

## Materials and Methods

### Participants

One hundred and seventy children participated in this experiment (mean age: 4.59 years, range: 4.48–4.90 years; 89 female). Of these, 145 children completed the non-symbolic numerical comparison, non-symbolic addition, symbolic math, and IQ assessments, and 75 of those children additionally completed the executive functions task. Twenty-five children did not complete one or more of the primary tasks of interest and were therefore excluded from all analyses. Participants were recruited as part of a larger longitudinal studying tracking the development of numerical cognition from infancy into the preschool years. Data was collected between October 2011 and July 2015, and data collection was stopped when the lab moved to a new institution out of state.

### Procedure

Children were tested in two separate sessions each lasting less than 1 h. During the first visit, children completed the symbolic math assessment, one session of the non-symbolic number comparison task, and the executive functions task. During the second visit, children completed the IQ assessment, a second session of the non-symbolic number comparison task, and a non-symbolic approximate arithmetic task. All children were tested individually in a quiet room, and the order of the tasks within each session was counterbalanced across participants. At each visit, parents gave written consent to a protocol approved by the local Institutional Review Board. Parents were compensated monetarily and children received a small toy.

#### Non-symbolic Numerical Comparison Task

On each trial, a touchscreen computer displayed two squares (8 cm × 9.5 cm) containing arrays of dots. Children were instructed to touch the square that contained more dots and to make this choice without counting. Arrays contained between 4 and 14 dots, and the numerical ratio between the arrays was 1:2, 2:3, 3:4, or 6:7. To control for non-numerical perceptual cues, the parameters of the arrays varied such that the smaller and larger numerical array each had the larger cumulative surface area on 50% of trials. All of the dots within a single array were homogenous in element size and color, and the color of each array varied randomly from trial to trial. Differential audio-visual feedback was provided after each trial, and children received a small sticker for each correct response to keep them engaged. Children performed practice trials until they made three consecutive correct responses or completed a maximum of ten trials. Children were tested with 60 trials in each session for a total of 120 trials at each time point. Each child’s ANS acuity was estimated using a psychophysical modeling technique (e.g., Halberda and Feigenson, 2008; Piazza et al., 2010) to calculate a Weber fraction (*w*) based on performance in the non-symbolic numerical comparison task. The resulting value of *w* represents the noise in each participant’s internal ANS representations, such that lower values of *w* correspond to less noise (i.e., higher ANS acuity).

#### Non-symbolic Approximate Addition Task

**FIGURE 1:**
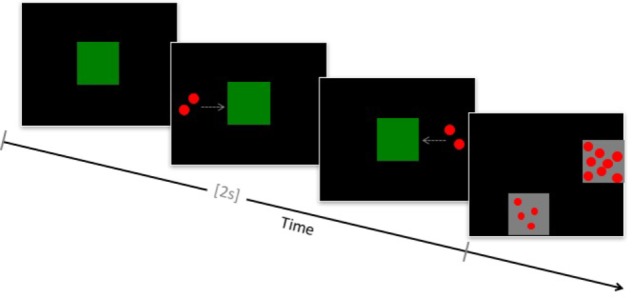
Schematic of the approximate arithmetic task.

This task was adapted from Cantlon and Brannon (2007). On each trial, children viewed an animation that consisted of an array of dots moving behind an occluding box, followed by a second array moving behind the same occluder (Figure 1). This animated arithmetic sequence lasted a total of 2000 ms. Children then saw two squares containing arrays of dots and were instructed to touch the array that contained the same number of dots as had moved behind the occluder box. The choice arrays remained on the screen until a decision was made. Correct and incorrect values differed by a 1:2 or 1:4 ratio. The specific problems presented were: 1+1 = 2, 4, or 8; 2+2 = 2, 4, or 8; 4+4 = 2, 4, or 8. Individual dot size varied across arrays but was homogenous within each array. Differential audiovisual feedback was provided after each trial, and children were rewarded with a small sticker for correct responses. Children performed practice trials until they made three consecutive correct responses or completed a maximum of ten trials. Children then completed a total of 42 test trials.

#### Executive Functions Task

The Day/Night task (Gerstadt et al., 1994) was used to assess executive functions. This task requires children to remember the relevant rule and to inhibit a prepotent verbal response. In the warm-up version, children were shown a card containing 16 sun and moon pictures in a pseudo-random order and instructed to say “day” for the sun pictures and “night” for the moon pictures as quickly as possible. Next, children were told they were going to play a silly version of the game that required saying the opposite picture names (“day” for the moon picture and “night” for the sun picture). They were then shown a new card with 16 sun and moon pictures and instructed to say the opposite picture names as quickly as possible without making mistakes. The total time and number of errors were combined into a single efficiency score (number of correct responses divided by total time).

#### Standardized Assessments

Children’s mathematical ability was assessed with the Test of Early Mathematics Ability (TEMA-3) (Ginsburg and Baroody, 2003), which consists of a series of verbally administered questions that assess age-appropriate counting ability, number-comparison facility, numeral literacy, and basic calculation skills. To assess general intelligence, children completed the two verbal (Guess What and Verbal Reasoning) and the two non-verbal subtests (Odd-Item Out and What’s Missing) of the Reynolds Intellectual Assessment Scales (RIASs) (Reynolds and Kamphaus, 2003). The verbal subtests are oral assessments of verbal knowledge and reasoning. The non-verbal subtests are visuospatial assessments of reasoning, spatial ability and general knowledge. The scores on these four subtests were combined to create a composite IQ score for each child.

## Results

Descriptive statistics and a correlation table for all measures of interest can be found in Tables 1, 2. The complete dataset can be found in the Supplementary Material.

**Table 1:** Descriptive statistics for all variables of interest.

Task	Measure	Mean *(SD)*
Non-symbolic numerical comparison	Accuracy (% correct)	79.45 (8.32)
	Weber fraction (*w*)	0.31 (0.15)
TEMA-3 (math achievement test)	Standardized score	111.57 (12.99)
RIAS (IQ test)	Standardized score	128.94 (16.15)
Day/Night (executive functions task)	Efficiency score	0.51 (0.26)
Approximate arithmetic	Accuracy (% correct)	77.27 (13.63)


**Table 2:** Correlation matrix of Pearson r values for all variables of interest.

	w	Symbolic math	IQ	Approximate addition	Executive functions
w	-	-0.27	-0.16	-0.22	-0.16
Symbolic math	-0.27	-	0.42	0.32	0.33
IQ	-0.16	0.42	-	0.21	0.18
Approximate addition	-0.22	0.32	0.21	-	0.40
Executive functions	-0.16	0.33	0.18	0.40	-


### Preliminary Analyses

First we performed planned paired *t*-tests to confirm that participants’ performance on the approximate addition task was modulated by ratio. Planned paired *t*-tests confirmed that children were both more accurate and responded more quickly on the 1:4 ratio trials compared to the 1:2 ratio trials in the approximate addition task [accuracy: *t*(144) = 8.63, *p* < 0.001; RT: *t*(144) = -4.90, *p* < 0.001], which suggests that this task engaged the ANS.

### Regression Analyses

**FIGURE 2:**
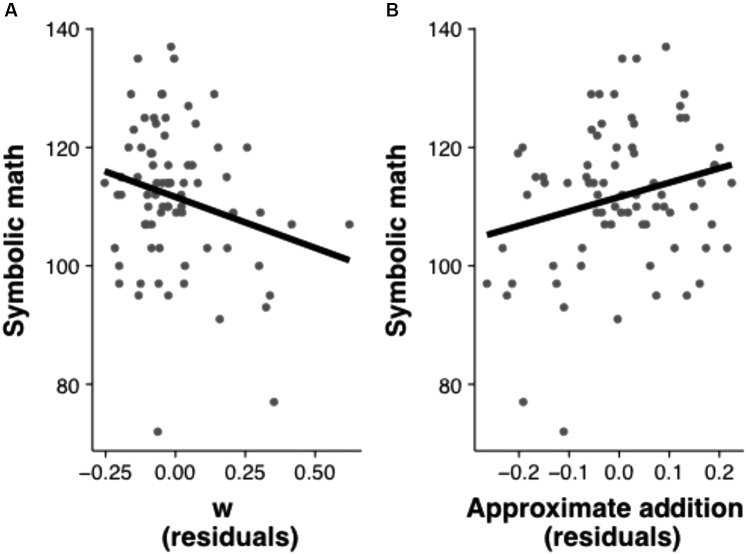
Scatterplots illustrating the relation between *w* and math achievement controlling for approximate addition, IQ, and executive functions **(A)** and the relation between approximate addition and math achievement controlling for *w*, IQ, and executive functions **(B)**.

In the first series of analyses, we used multiple regression models to investigate the unique variance contributed by each of our measures of interest (Table 3). The first model (Model 1) examined the variance in symbolic math achievement predicted by ANS acuity (indexed by *w*), ANS manipulability (indexed by approximate addition performance), and IQ. This model revealed that all factors contributed significant variance (β*_w_* = -0.24, *p* < 0.05, β*_ApproxAdd_* = 0.27, *p* < 0.005, β*_IQ_* = 0.33, *p* < 0.001; all betas are standardized). We next ran a second model that included the executive functions task for the subset of participants who completed this task (Model 2). In this model, the original predictors all remained significant, but the executive functions task did not explain significant additional variance (β*_w_* = -0.24, *p* < 0.05, β*_ApproxAdd_* = 0.27, *p* < 0.05, β*_IQ_* = 0.33, *p* < 0.001, β*_EF_* = 0.20, *p* = 0.054). These analyses suggest that the acuity and manipulability of the ANS each contribute unique variance to preschooler’s early symbolic math skills that is not accounted for by IQ or executive functions (Figure 2).

**Table 3:** Regression models predicting symbolic math achievement.

	Model 1		Model 2	
*R*^2^	0.247		0.383	
*F*-statistics	*F*(3,141) = 16.72		*F*(4,70) = 12.48	
*p*-statistics	*p* < 0.001		*p* < 0.001	
*N*	145		75	

**Predictor**	**β_Adjusted_**	***p***	**β_Adjusted_**	***p***

ANS acuity	-0.179	0.018	-0.239	0.014
ANS manipulability	0.237	0.002	0.267	0.011
IQ	0.325	0.332	<0.001	
Executive functions	–	–	0.196	0.054


### Mediation Analyses

**FIGURE 3:**
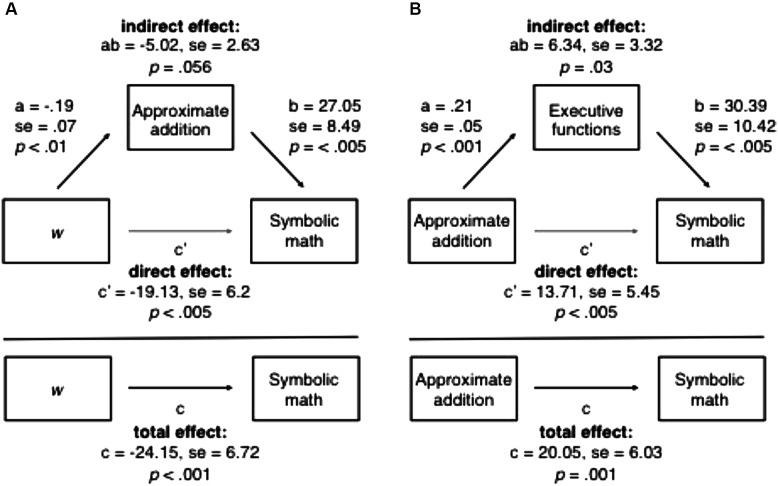
Mediation models assessing whether **(A)** approximate addition mediates the relation between *w* and symbolic math and **(B)** whether executive functions mediate the relation between approximate addition and math. Note that in both cases the direct effect remains significant after accounting for the variance attributable to the mediator, indicating that the mediation is not complete.

Next we used structural equation modeling to determine whether the relation between ANS acuity and symbolic math achievement in mediated by ANS manipulability (Figure 3). This method enables us to directly test which portion of the relation between ANS acuity and symbolic math can be accounted for by ANS manipulability. The mediation analysis was performed using the lavaan package in R (Rosseel, 2012). The results of the mediation analyses indicate that the direct effect (c′ = -19.13, *SE* = 6.2, *p* < 0.005) is significant whereas the indirect effect is not (ab = -5.02, *SE* = 2.63, *p* = 0.056). Because the direct effect remains significant after accounting for the variance contributed by the mediator and the mediation path is not significant, this suggests that ANS manipulability does not mediate the relation between ANS acuity and symbolic math achievement. Rather, ANS acuity and ANS manipulability are each making independent contributions to symbolic math achievement in preschoolers.

We also tested whether executive functions mediate the relation between approximate arithmetic performance and symbolic math. This model indicated that both the direct effect (c′ = 13.71, *SE* = 5.43, *p* = 0.01) and the indirect effect are significant (ab = 6.34, *SE* = 2.72, *p* = 0.02). Because the direct effect from approximate addition to symbolic math achievement remains significant after accounting for executive functions, this result suggests that executive functions do not fully mediate the relation between approximate arithmetic performance and math. Together, the results of these mediation analyses are consistent with the multiple regression analyses in suggesting that approximate arithmetic is contributing unique variance to children’s symbolic math achievement that is not shared with ANS acuity or executive functions.

## Discussion

The goal of the present research was to investigate the mechanisms by which approximate number representations contribute to preschoolers’ emerging symbolic math capabilities. Consistent with previous studies, we found that individual differences in the precision of the ANS are related to symbolic math achievement in preschool-aged children (e.g., Libertus et al., 2011; Starr et al., 2013b; vanMarle et al., 2014). In addition, we found that children’s proficiency with manipulating ANS representations contributed additional unique variance to their symbolic math achievement that was not accounted for by ANS acuity, IQ, or executive functions. Together, these results suggest that both the acuity and manipulability of the ANS influence children’s early math performance.

The majority of studies relating the ANS to symbolic math have focused on individual differences in the acuity of approximate number representations. However, the present results suggest that the manipulability of these representations is a second mechanism by which the ANS influences symbolic math. Although both non-symbolic numerical comparison and approximate arithmetic tasks require representing approximate numerical quantities, approximate addition additionally requires the manipulation of those quantities. Previous studies in infants, young children, and monkeys, all of whom have no understanding of symbolic arithmetic, demonstrate that the ANS supports arithmetic operations (McCrink and Wynn, 2004; Barth et al., 2005; Cantlon and Brannon, 2007). Like symbolic arithmetic, successful approximate arithmetic requires not just representing numerical quantities but also combining them to form summed quantity. Therefore, approximate arithmetic may provide an intuitive basis for the acquisition of symbolic arithmetic principles. Consistent with this view, we found that approximate arithmetic ability in 4.5-year-olds was a significant predictor of performance on a standardized assessment of symbolic math ability. Further, approximate arithmetic ability predicted unique variance in symbolic math scores that was not accounted for by ANS acuity, IQ, or executive functions. This result suggests that although there is a correlation between the acuity of children’s ANS representations and their ability to manipulate those representations, these two factors make independent contributions to children’s emerging math abilities.

Because approximate addition requires mental manipulation, it likely places a greater demand on executive functions, including working memory and updating, compared to non-symbolic numerical comparison. Given the well documented link between executive functions and math achievement in children (e.g., Bull and Scerif, 2001; St Clair-Thompson and Gathercole, 2006; Bull and Lee, 2014), one potential alternate explanation of our findings might be that the apparent link between ANS manipulability and symbolic math is actually a link between executive functions and math. However, there are multiple reasons to believe that this is not the case. First, we found that approximate addition performance was a significant predictor of math achievement even after controlling for performance on an independent executive functions task, and we found that executive functions did not mediate the relation between approximate arithmetic performance and symbolic math. Training studies in adults and children provide additional evidence that approximate arithmetic taps a cognitive skill that is separable from executive functions. These studies have found that training approximate arithmetic leads to greater improvement in symbolic arithmetic performance than does working memory training, and that approximate arithmetic training does little to improve working memory or executive functions (Park and Brannon, 2014; Park et al., 2016).

However, executive functions are a multifaceted construct (Miyake et al., 2000; Lehto et al., 2003), and we are limited in the conclusions we can draw from the use of a single executive functions task. In the present study, we used the Day/Night task (Gerstadt et al., 1994) to measure executive functions, which is similar to the task that has been used in previous studies investigating whether inhibitory control mediates the link between ANS acuity and symbolic math (Fuhs and Mcneil, 2013; Gilmore et al., 2013). This task requires both working memory (to maintain and apply the current role) and inhibitory control (to inhibit the prepotent verbal response). However, it is possible that if we had used a separate assessment of working memory, we would have found a closer link to our approximate arithmetic task. In particular, it would be interesting to test how spatial attention interacts with approximate addition performance, given the relation between spatial attention and math achievement (Bull et al., 2008; Geary, 2011). Critically, the current results are not inconsistent with the view that executive functions contribute to successful approximate arithmetic, and disentangling the relation between approximate arithmetic and executive functions will be an important direction for future research.

In contrast to a previous finding (Pinheiro-Chagas et al., 2014), we did not find that approximate addition performance fully mediates the relation between ANS acuity and symbolic math. Although differences in the non-symbolic comparison and approximate addition tasks used may have contributed to these inconsistent results, another possible explanation is the difference in the ages of the participants. The children in the Pinheiro-Chagas et al. (2014) study averaged 10 years of age, whereas the participants in the present study were only four. This age difference means that the children have vastly different knowledge of and experience with symbolic arithmetic. The relation between ANS acuity and symbolic math is not static with age: two recent meta-analyses have shown that the correlation between ANS acuity and symbolic math performance is strongest in young children and decreases with age (Fazio et al., 2014; Schneider et al., 2017). Therefore, it is also likely that the link between ANS manipulability and symbolic math changes with age, and this is an important area for future research.

A limitation of these data is that our approximate addition task only used numerosities between 1 and 8, which means that many of the numerosities fall within the subitizing range. However, the presence of ratio effects for both accuracy and reaction times suggests that children were not relying on subitizing to solve the addition problems. In addition, due to the speed of the addition animation, it is unlikely that children were counting the items or using a symbolic labeling strategy, and such strategies were actively discouraged. Previous work in human adults (Cordes et al., 2001; Hyde and Wood, 2011), infants (Wynn et al., 2002; Izard et al., 2008; Starr et al., 2013a), and non-human primates (Brannon and Terrace, 1998) demonstrates that the ANS can be engaged to represent both small and large numerosities. Notably, Hyde and Spelke (2011) previously suggested that stimulus complexity may predict whether small numerosities are represented by subitizing or parallel individuation versus the ANS; when stimuli are more simple, parallel individuation processes may be recruited, but when stimuli are more complex, the ANS may be recruited. This proposal can explain why infants are able to engage the ANS and succeed in discriminating two versus four elements when the displays are dynamic (Wynn et al., 2002; Starr et al., 2013a), yet fail to do so in other situations (Feigenson and Carey, 2003; Xu, 2003). The approximate addition task in the present experiment involved animated displays of moving arrays of dots, which is a situation that is likely to engage the ANS. In addition, children’s approximate addition performance was ratio-dependent, meaning that accuracy was greater for trials with a 1:4 ratio compared to a 1:2 ratio. This pattern of performance, which is also seen when adults and monkeys perform approximate addition using a very similar task (Cantlon and Brannon, 2007), suggests that performance on the task is supported by the ANS. Given that approximate addition performance contributes unique variance to symbolic math achievement after controlling for ANS acuity, IQ, and executive functions, it is parsimonious to conclude that our approximate addition task is tapping a cognitive skill not indexed by these other measures, and we believe this skill is the manipulation of approximate quantities. However, additional studies using approximate addition tasks with larger set sizes are needed to corroborate this conclusion.

## Conclusion

The ANS endows young children with a robust sense of quantity prior to beginning formal mathematics instruction. Although many studies have provided evidence for a correlation between the fidelity of the ANS and symbolic math achievement, there remain key open questions concerning the mechanisms underlying this relation. In the present study, we provide evidence that the acuity and manipulability of the ANS have separable influences on preschoolers’ early symbolic math proficiency. In particular, the influence of ANS manipulability may stem from its ability to support arithmetic operations. The shared demand for manipulating quantities may form a conceptual bridge between non-symbolic and symbolic arithmetic. Our findings therefore suggest a nuanced relation between approximate number representations and symbolic math achievement in which multiple features of the ANS contribute to the emergence of symbolic math ability in young children. In light of these results, interventions designed to target one or both of these pathways may be differentially beneficial for children depending on their level of symbolic number knowledge and mathematical proficiency.

## Ethics Statement

The study and protocol were reviewed and approved by the Duke University’s Institutional Review Board. Written informed consent was obtained from the guardians of all participants and assent was obtained from all participants.

## Author Contributions

AS and EB conceived and planned the experiment. AS and RT collected the data. AS performed the analyses. All authors discussed the results and contributed to the final manuscript.

## Conflict of Interest Statement

The authors declare that the research was conducted in the absence of any commercial or financial relationships that could be construed as a potential conflict of interest.

## Footnotes

1 The executive functions task was added to the battery after a 2013 paper (Gilmore et al., 2013) suggested that the link between ANS acuity and symbolic math achievement may be a by product of the link between inhibitory control and symbolic math achievement.
